# Expression levels of atherosclerosis-associated miR-143 and miR-145 in the plasma of patients with hyperhomocysteinaemia

**DOI:** 10.1186/s12872-017-0596-0

**Published:** 2017-06-20

**Authors:** Kejian Liu, Saiyare Xuekelati, Yue Zhang, Yin Yin, Yue Li, Rui Chai, Xinwei Li, Yi Peng, Jiangdong Wu, Xiaomei Guo

**Affiliations:** 1 Department of Cardiology, Tongji Hospital, Tongji Medical College, Huazhong University of Science and Technology, Wuhan, 430030 China; 2 Department of Cardiology, the First Affiliated Hospital, Shihezi University School of Medicine, Shihezi, Xinjiang China; 3 The Key Laboratory of Xinjiang Endemic and Ethnic Diseases, Shihezi University, Shihezi, Xinjiang 832000 China

**Keywords:** miR-143, miR-145, Hyperhomocysteinaemia, Atherosclerosis, Correlation

## Abstract

**Background:**

An elevated level of homocysteine (Hcy) in the blood is designated hyperhomocysteinaemia (Hhcy) and is regarded as a strong risk factor for the development of atherosclerosis (ATH), although the association remains controversial. Considered to be essential gene expression regulators, micro-RNAs (miRNAs) modulate cardiovascular disease development and thus can be regarded as potential biomarkers and therapeutic targets in atherosclerosis. The aim of the current study is to investigate the expression levels of atherosclerosis-associated miR-143 and miR-145 in Hhcy patients and predict the progress of atherosclerosis in Hhcy patients.

**Methods:**

A total of 100 participants were enrolled and included normal control subjects (NC = 20), hyperhomocysteinaemia alone subjects (Hhcy = 25), hyperhomocysteinaemia and carotid artery atherosclerosis combined subjects (Hhcy + ATH = 30) and patients with standalone carotid artery atherosclerosis (ATH = 25). Plasma Hcy, supplementary biochemical parameters and carotid artery ultrasonography (USG) were measured in all participants. MicroRNA expression levels in the peripheral blood were calculated by real-time reverse transcription-polymerase chain reaction (qRT-PCR). The correlations of miR-143 and miR-145 with Hcy, blood lipid parameters and carotid artery atherosclerotic plaques were evaluated using Pearson’s correlation coefficients. Receiver operating characteristic (ROC) curve analyses were performed to evaluate the capacities of miR-143 and miR-145 for the detection of Hhcy and atherosclerosis patients.

**Results:**

MiR-143 and miR-145 exhibited trends towards significance with stepwise decreases from the NC to Hhcy groups and then to the Hhcy + ATH and ATH groups. Similar results were observed in the carotid artery plaque group (Hhcy + ATH and ATH grups) compared with the no-plaque group (NC and Hhcy groups). The miR-143 expression level exhibited significant negative correlations with Hcy, total cholesterol (TC) and low-density lipoprotein cholesterol (LDL-c). The miR-145 expression level exhibited significant negative correlations with Hcy, TC, triglyceride (TG) and LDL-c. MiR-143 and miR-145 exhibited the greatest area under the curves (AUCs) (0.775 and 0.681, respectively) for the detection of every Hhcy patient, including those in the Hhcy and Hhcy + ATH groups, from among all subjects.

**Conclusion:**

The results indicated that the levels of atherosclerosis-associated circulating miR-143 and miR-145 are linked to Hhcy. MiR-143 may be used as a potential non-invasive biomarkers of Hhcy and thus may be helpful in predicting the progress of atherosclerosis in Hhcy patients.

**Electronic supplementary material:**

The online version of this article (doi:10.1186/s12872-017-0596-0) contains supplementary material, which is available to authorized users.

## Background

Homocysteine (Hcy) is an intermediate substance that is formed in the metabolic pathway of cysteine and methionine. Hyperhomocysteinaemia (Hhcy) is regarded as an emerging risk factor for the development of atherosclerosis and a variety of other cardiovascular diseases, such as coronary artery disease (CAD), hypertension, stroke, etc. [1–3]*.* Recent studies have demonstrated that homocysteine initiates an inflammatory response in vascular smooth muscle cells (VSMCs) and triggers the proliferation and migration of VSMCs [4, 5]. Moreover, it is well established that VSMC proliferation and migration play fundamental roles in the development of atherosclerosis [6, 7]. We hypothesize that Hhcy may induce the process of atherosclerosis. Although several studies have implicated Hhcy in atherosclerosis, the exact mechanism is not entirely understood.

MicroRNAs (miRNAs) are single-stranded noncoding RNA molecules of 22 nucleotides. miRNAs inhibit mRNA translation by interacting with the 3′ untranslated region (UTR) [8–10]. To determine the ability of atherosclerosis-associated microRNAs to predict the presence of atherosclerosis in Hhcy patients, miRNA databases (i.e., the TargetScan database, http://www.targetscan.org/ and miRBase, http: //http://www.mirbase.org/) [11] and other relevant literature were searched. MiR-143 and miR-145 were confirmed to be critical factors in the development of atherosclerosis [12]. Circulating miR-143 is critical for the regulation of VSMC phenotypes because it promotes differentiation and prevents the proliferation of VSMCs [13, 14]. Because miR-145 is the most abundant miRNA in the vascular wall, it plays a crucial role in differentiation of VSMCs and inhibits their proliferation [15, 16].

There is accumulating evidence demonstrating that miRNAs are a key factor in the development of atherosclerosis [9, 13, 17, 18] and other cardiovascular diseases. Down-regulation of miR-143/−145 has been predominantly expressed in VSMCs and play a role in several cardiovascular diseases, such as hypertension and coronary artery disease (CAD). Recent clinical fndings have demonstrated that the expression levels of miR-143/−145 were decreased in essential hypertension patients compared with healthy subjects [19, 20]. MiR-145 contributes to the pathogenesis of hypertension, as it mediates stretch-induced differentiation of VSMCs [21, 22]. These findings highlight the importance of miR-143/−145 as potential biomarkers for cardiovascular diseases. Vascular smooth muscle cells play an important role in plaque stabilization, particularly in the progression of atherosclerosis [23]. Gain and loss of function of VSMC-enriched miR-143/−145 in vivo results in reduced proliferation, which consequently limits neointima formation in vascular injury models. This leads to the assumption that the miR-143/−145 are inevitable for the pro-proliferative response of VSMCs to injury [24, 25]. This suggests that downregulation of miR-143/−145 may contribute to atherogenesis. MiR-143/−145 are relatively specific for VSMCs and thus are closely correlated with cardiovascular diseases such as atherosclerosis, hypertension and CAD.

However, the exact correlates of the expressions of atherosclerosis-associated microRNAs in Hhcy patients have yet to be fully elucidated. To determine whether these two miRNAs are related to hyperhomocysteinaemia and thus to the development of atherosclerosis, a study was conducted that involved a comparison of their expression levels in the plasma of hyperhomocysteinaemia alone patients and patients with both hyperhomocysteinaemia and carotid atherosclerosis and to further examine whether miRs-143/145 are related directly to hyperhomocysteinaemia and the development of carotid atherosclerotic plaques in humans, the associations with hyperhomocysteinaemia and atherosclerosis were re-analysed from a different perspective.

## Methods

### Research subjects

For this study, hyperhomocysteinaemia was defined as a plasma Hcy level above 15 μm/L [26]. Carotid artery atherosclerosis was determined by carotid artery ultrasonography (USG), which was used to determine the presence of plaque formation, which in turn was used to categorize the subjects into plaque and no-plaque groups. Approximately 310 newly diagnosed Hhcy patients were screened in the Department of Cardiovascular Diseases of the First Affiliated Hospital of Shihezi University Medical College from January 2014 to December 2015. Patients with histories of hypertension, ischaemic heart disease, diabetes mellitus, chronic liver diseases, chronic renal diseases and excessive drinking plus smoking were excluded. Ultimately, only 167 Hhcy patients were included. Of these, 30 Hhcy + ATH patients and 25 Hcy standalone patients were selected. The two groups were matched for age, sex, body mass index (BMI). Additionally, 25 ATH and 20 NC subjects were selected and were matched to the Hhcy + ATH group for age, sex and BMI. These subjects were extracted from 800 individuals who were already receiving health check-ups at the physical examination centre of the First Affiliated Hospital of Shihezi University Medical College during the same period.

### Carotid artery ultrasonography to determine plaque formation

All patients underwent carotid artery ultrasonography (USG) to determine plaque formation. The patients were asked to lie down one by one in a semi-dark room in the supine position with their neck slightly extended and rotated away from the imaging transducer. Both the right and left carotid arteries and the bifurcation were visualized by an experienced radiologist via an ultrasonography device (Hitachi) using a 9-MHz linear probe. According to the study criteria, the absence of atherosclerotic plaque was considered “normal”, and positive results were indicative of atherosclerotic plaque [27].

### Biochemical assays

Whole blood samples were collected in Ethylene Diamine Tetraacetic Acid (EDTA) tubes from each subject in the morning after an 8-h fast. Plasma Hcy, TG, TC, high-density lipoprotein cholesterol (HDL-c), LDL-c, apolipoprotein A (ApoA), apolipoprotein B (ApoB), apolipoprotein A/B (Apo(A/B)), fasting blood glucose (FBG), alanine aminotransferase (ALT), aspartate aminotransferase (AST) and uric acid (UA) and serum creatinine (Cre) were measured using a Hitachi 7600 automated biochemistry analyser at the First Affiliated Hospital of Shihezi University Medical College.

### RNA isolation and microRNA calculation

Whole blood samples were collected in tubes containing EDTA in the morning after 8 hs of fasting. The samples were immediately centrifuged at 3000 rpm for 10 mins at room temperature. After the separation phase, the plasma was collected, divided into two aliquots and frozen at −80 °C for later RNA isolation.

Total RNA, containing small RNA, was extracted from plasma using Trizol -Reagent (Tiangen, Biotech, Beijing, China), according to the manufacturer’s protocol. For miRNA qPCR, prior RT was performed using miRNA FastKing RT Kit (Tiangen Biotech Co, Ltd., Beijing, China, no. KR160815). For RT, 1 μg of RNA containing miRNA was polyadenylated by poly (A) polymerase and then reverse transcribed to cDNA. The cDNA (2 μl) then served as the template and was added to 1 μl primers for SYBR Green real-time PCR using miRcute Plus miRNA qPCR Detection Kit (Tiangen Biotech Co, Ltd., Beijing, China, no. FP160303). The sequence of the miR-143 specific forward primer was 5′-TGAGATGAAGCACTGTAGCTC-3′and the reverse primer was 5′-GCTGTCAACATACGCTACGTAACG-3′. The sequence of the miR-145 specific forward primer was 5′-GTCCAGTTTTCCCAGGAATCCCT-3′and the reverse primer was 5′-GCTGTCAACATACGCTACGTAACG-3′. Following the illustration in previous studies, measurements of microRNA levels were performed by quantitative RT-qPCR using cel-miR-54 as normalization control [28, 29]. The sequence of the cel-miR-54 forward primer was 5′-CCGCCCTACCCGTAATCTTCATAA-3′ and the reverse primer was 5′-GTGCAGGGTCCGAGGT-3′. The RT reaction was performed at first at 37 °C for 30 min, followed by at 42 °C for 30 min and finally at 75 °C for another 5 min. The PCR reaction was performed initially at 95 °C for 3 min, followed by repeated 40 cycles at 95 °C for 10s and latter at 60 °C for 30s. The comparative Ct method (ΔCt) was exploited to calculate the relative expression level of miR. The relative expression of each miRNA after normalization to cel-miR-54 is displayed as 2^- (Ct [miRNA] -Ct[cel-miR-54])^ [30].

### Statistical analysis

SPSS software (SPSS, Inc., Chicago, USA) for Windows version 20.0, STATA statistics software (Version 12.0, Stata corporation, College Station, Texas 77, 845 USA) and GraphPad Prism 5.0 software (GraphPad Software, Inc., La Jolla, CA, USA) were used for the statistical analysis. All data were subjected to a normality test (Kolmogorov-Smirnov). For the baseline characteristics of the patients and controls, the continuous variables were summarized as the means ± the SDs, and the discrete variables were summarized as counts and proportions. For the normally distributed data, one-way analysis of variance (ANOVA) and multiple comparison (LSD) tests were applied. The Kruskal-Wallis test was performed to compare the data that were not normally distributed. The significance of the microRNA level differences between the carotid artery plaque group and the no-plaque group were assessed with independent sample Student’s t-tests. The χ [2] test was used to compare the gender distributions. Pearson correlations were used to explore the relationships of the miRNAs with Hcy, the lipid parameters, and the carotid atherosclerotic plaque value. Receiver operating characteristic (ROC) curve analyses were performed to evaluate the capacities of miR-143 and miR-145 to detect Hhcy and atherosclerosis patients. Combined diagnostic accuracy of circulating miRNAs both in the all Hhcy patients and all atherosclerosis patients by using STATA statistics software. *P* < 0.05 was regarded as statistically significant.

## Results

### Significant differences in the clinical features of the four groups

**Fig. 1:**
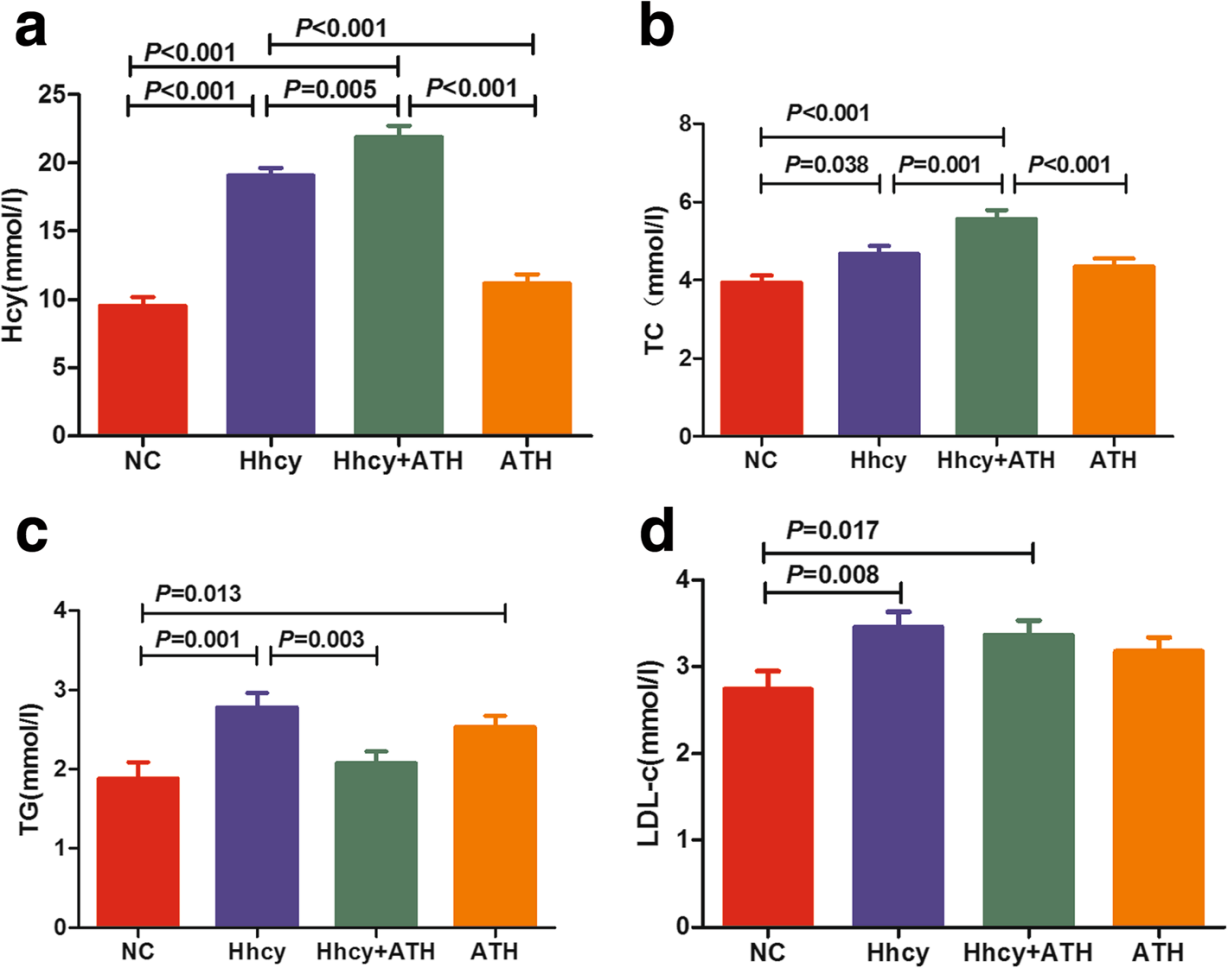
Multiple comparisons of baseline characteristics (Hcy, TC, TG and LDL-c) among NC, Hhcy, Hhcy + ATH and ATH subjects. Data are shown as the mean ± SD. *P* values were generated by one-way ANOVA test followed by the LSD post hoc multiple comparisons test. *P* < 0.05 or *P* < 0.001 was considered significant

The basic characteristics and clinical features of the studied groups are provided in Table 1. Hcy, TG, TC and LDL-c exhibited differences between the groups (*P* < 0.001). No significant differences were found in the other clinical factors, which included the gender distribution, BMI, age, HDL-c, etc. (Table 1). LSD post hoc multiple comparison tests revealed that the serum TC and LDL-c levels in the Hhcy and Hhcy + ATH groups were significantly higher than that in the NC group. Additionally, the serum TC levels in the Hhcy + ATH group were higher than those in the Hhcy and ATH groups, whereas no differences in serum LDL-c levels were found between the Hhcy + ATH, Hhcy, and ATH subjects. The TG level in the Hhcy group was higher than those in the Hhcy + ATH and NC groups (Fig. 1).

**Table 1:** Significant differences in clinical features among the four groups

	NC	Hhcy	Hhcy + ATH	ATH	*P*-value
Males/females	10/10	14/11	20/10	15/10	0.682
Age (years)	48.00 ± 4.44	47.16 ± 5.50	49.00 ± 5.47	46.92 ± 5.20	0.445
BMI (kg/m2)	24.13 ± 2.84	23.47 ± 2.30	25.03 ± 3.58	23.13 ± 2.68	0.080
TC (mmol/l)	3.94 ± 0.79	4.60 ± 0.99	5.56 ± 1.26	4.34 ± 1.04	<0.001**
TG (mmol/l)	1.12 ± 0.07	1.37 ± 0.04	1.45 ± 0.09	1.41 ± 0.11	0.001**
HDL-c (mmol/l)	1.43 ± 0.55	1.58 ± 0.63	1.59 ± 0.57	1.82 ± 0.66	0.176
LDL-c (mmol/l)	2.74 ± 0.91	3.46 ± 0.88	3.36 ± 0.93	3.18 ± 0.81	0.043*
ApoA(g/l)	1.32 ± 0.22	1.39 ± 0.27	1.39 ± 0.41	1.47 ± 0.31	0.480
ApoA/B	1.51 ± 0.42	1.57 ± 0.5	1.42 ± 0.45	1.53 ± 0.43	0.671
ApoB(g/l)	0.96 ± 0.25	0.93 ± 0.19	1.01 ± 0.28	1.01 ± 0.31	0.580
FBG (mmol/l)	5.06 ± 0.74	4.93 ± 0.55	5.07 ± 0.5	4.93 ± 0.59	0.722
ALT (U/l)	22.8 ± 13.02	19.8 ± 10.17	27.0 ± 12.8	20.48 ± 10.86	0.100
AST (U/l)	20.25 ± 10.17	20.84 ± 10.02	20.57 ± 12.36	21.2 ± 10.73	0.993
UA (umol/l)	255.65 ± 63.2	302.96 ± 105.9	258.9 ± 114.2	224.12 ± 106.1	0.795
Cre (umol/l)	73.68 ± 22.12	75.72 ± 24.12	71.3 ± 16.16	69.88 ± 19.84	0.754
Hcy (umol/l)	9.53 ± 2.99	19.07 ± 2.85	21.90 ± 4.69	11.16 ± 2.43	< 0.001**

Continuous and categorical variables data were expressed as mean ± SEM. The statistical *P* value was generated by the one-way ANOVA test or Kruskal-Wallis test. χ^2^ test was employed to compare gender distribution. *P < 0.05 or **P < 0.001 was considered significant

### MiR-143 and miR-145 were easily detected in the serum samples of all subjects

**Fig. 2:**
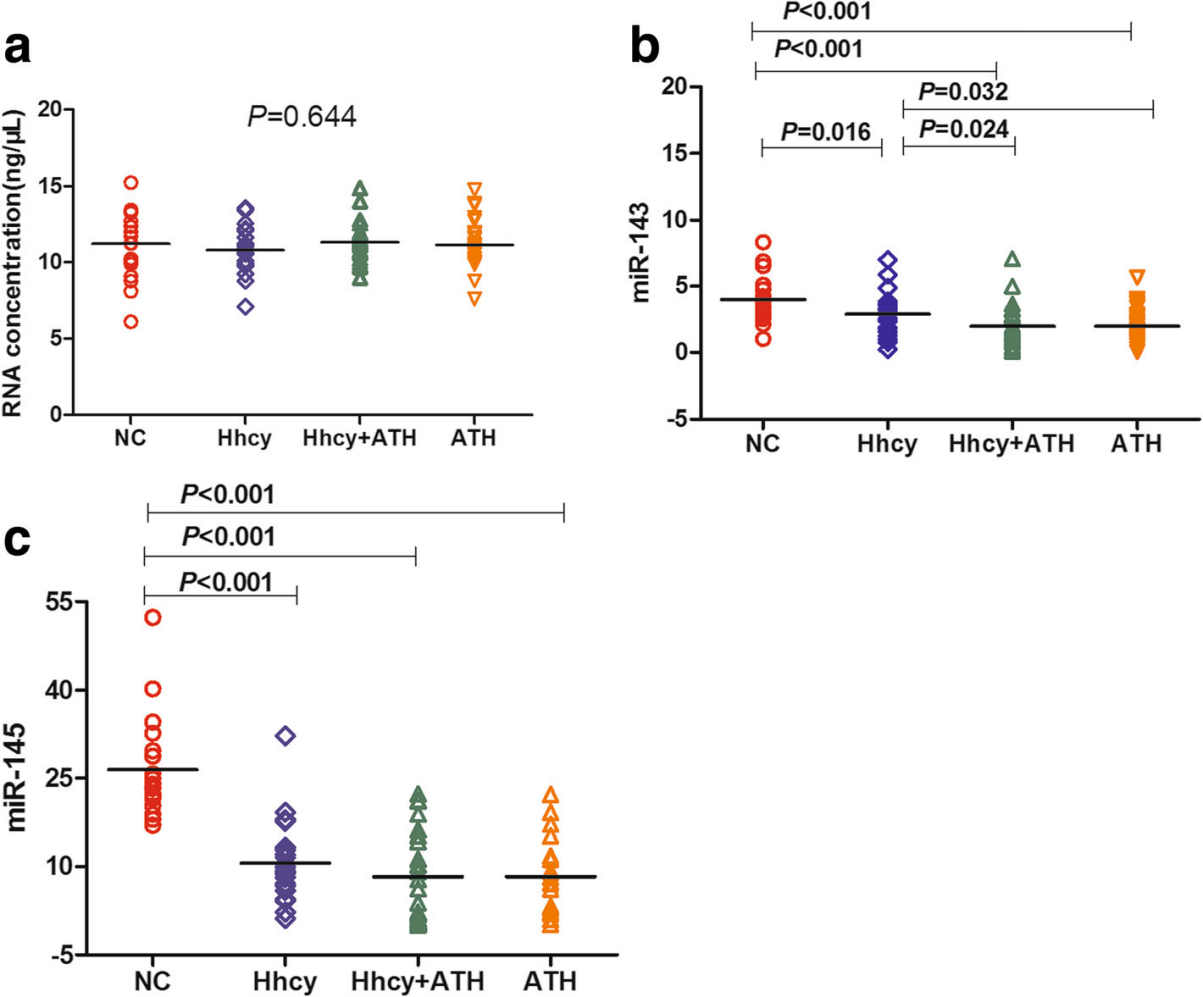
**a** Concentration of all RNA samples has no significance difference among four groups. **b**, **c** The relative expression levels of miR-143 and miR-145 in the NC, Hhcy, Hhcy + ATH and ATH groups. The *horizontal lines* indicate the mean. *P* values were generated by one-way ANOVA test followed by the LSD post hoc multiple comparisons test. *P* < 0.05 or *P* < 0.001 was considered significant

Before using ΔΔCt for relative gene expression, we checked the efficiency of target genes and reference gene and amplification efficiency is consistent. MiR-143 and miR-145 were stably measured in the serum samples of all subjects. No significant differences were discovered in the total RNA concentrations between the four groups (NC = 11.22 ± 2.14 ng/μl; Hhcy = 10.78 ± 1.41 ng/μl; Hhcy + ATH = 11.32 ± 1.23 ng/μl, and ATH = 11.15 ± 1.61 ng/μl) (Fig. 2a).

As displayed in Fig. 2b and c, both miR-143 and miR-145 exhibited significant trends towards stepwise decreases from the NC (3.99 ± 1.71 and 26.47 ± 8.47, respectively) to the Hhcy (2.89 ± 1.52 and 10.67 ± 5.26, respectively) to the Hhcy + ATH (1.96 ± 1.44 and 8.31 ± 7.21, respectively) and to the ATH (1.97 ± 1.35 and 8.35 ± 6.64, respectively) groups. No significant difference was found between the Hhcy and Hhcy + ATH subjects.

### Expression levels of miR-143/145 in the carotid plaque group versus the no plaque group

**Fig. 3:**
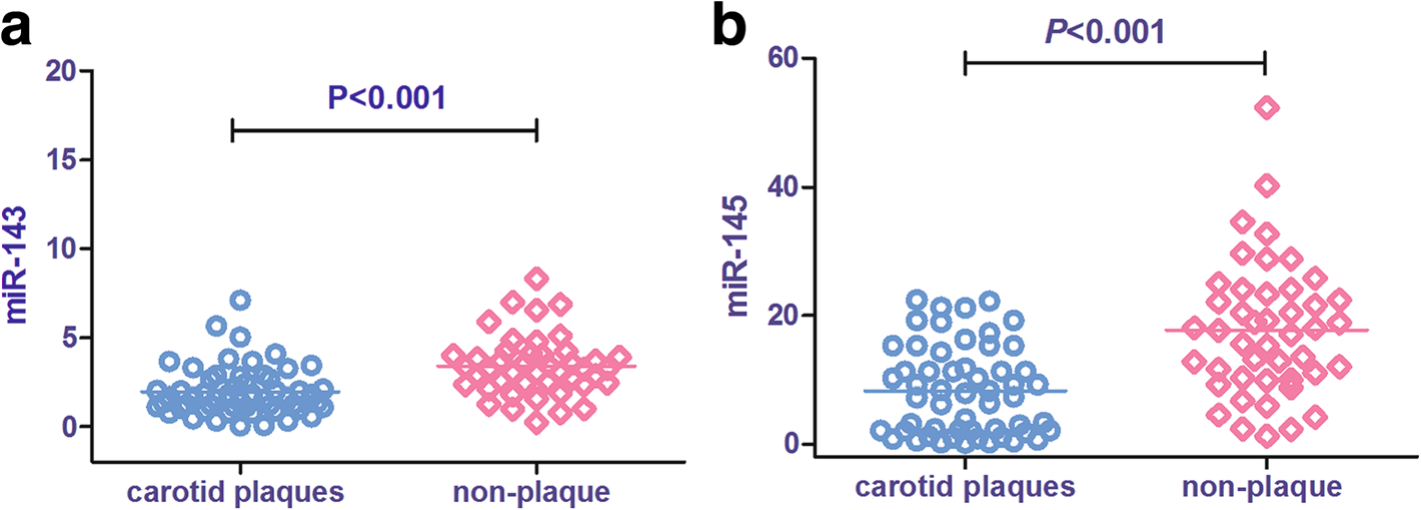
MiR-143 and miR-145 levels in carotid plaques group and non-plaque group. *P* < 0.001 was considered significant. Carotid plaques, all objects with carotid plaque (Hhcy + ATH and ATH group); Non-plaque, all objects without carotid plaque (NC and Hhcy group)

To better understand the pathophysiology of the atherosclerotic process, we compared the expressions of the candidate miRNAs in the atherosclerotic plaque and normal arteries. Among all subjects, 55 patients had carotid artery plaques (35 men and 20 women; mean age = 48.15 ± 5.36 years), and 45 patients had no plaques (24 men and 21 women, mean age = 47.53 ± 5.02 years). Lower levels of the expressions of miR-143 and miR-145 (1.97 ± 1.39 vs 3.38 ± 1.69 and 8.33 ± 6.90 vs 17.69 ± 10.45; *P* < 0.001) were observed in the carotid artery plaque group compared with the no-plaque group (Fig. 3).

### Correlations of miR-143 and miR 145 with Hcy and the lipid parameters

Baseline data about the correlations of miR-143/miR-145 with Hcy and the lipid parameters are presented in Table 2 and the Additional files (Additional file 1: Figure S1, Additional file 2: Figure S2, and Additional file 3: Figure S3). Pearson’s correlation analyses demonstrated that Hcy was positively correlated with TC (*r* = 0.299, *P* = 0.003) and LDL-c (*r* = 0.279, *P* = 0.005). The miR-143 expression level exhibited negative correlations with Hcy (*r* = −0.214, *P* = 0.032), TC (*r* = −0.390, *P* < 0.001) and LDL-c (*r* = −0.608, *P* < 0.001). The miR-145 expression level also exhibited negative correlations with Hcy (*r* = −0.347, *P* < 0.001), TC (*r* = −0.468, *P* < 0.001), TG (*r* = −0.594, *P* < 0.001) and LDL-c (*r* = −0.219, *P* = 0.028). However, no correlation was found between miR-143 and TG. Similarly, no correlation of miR-143/miR-145 with ApoA, ApoB, ApoA/B, age, BMI and no association of miR-143/miR-145 with gender, were found (*P* > 0.05).

**Table 2:** Correlations between miRNAs and Hcy, lipid profiles in all groups

		TC	LDL-c	TG	miR-143	miR-145
Hcy	r	0.299	0.279	0.053	−0.214	−0.347
	P	0.003*	0.005*	0.604	0.032*	<0.001**
TC	r		0.337	0.080	−0.390	−0.468
	P		0.001*	0.429	<0.001**	<0.001**
LDL-c	r			0.066	−0.608	−0.594
	P			0.513	<0.001**	<0.001**
TG	r				−0.123	−0.219
	P				0.223	0.028*
miR-143	r					0.690
	P					<0.001**

Pearson’s correlation correlations were presented as correlation coefficients (r) and significance (*P*), *P < 0.05 or **P < 0.001 was considered significant

### ROC curves for miR-143/145 for the definite detection of Hhcy and ATH patients

**Fig. 4:**
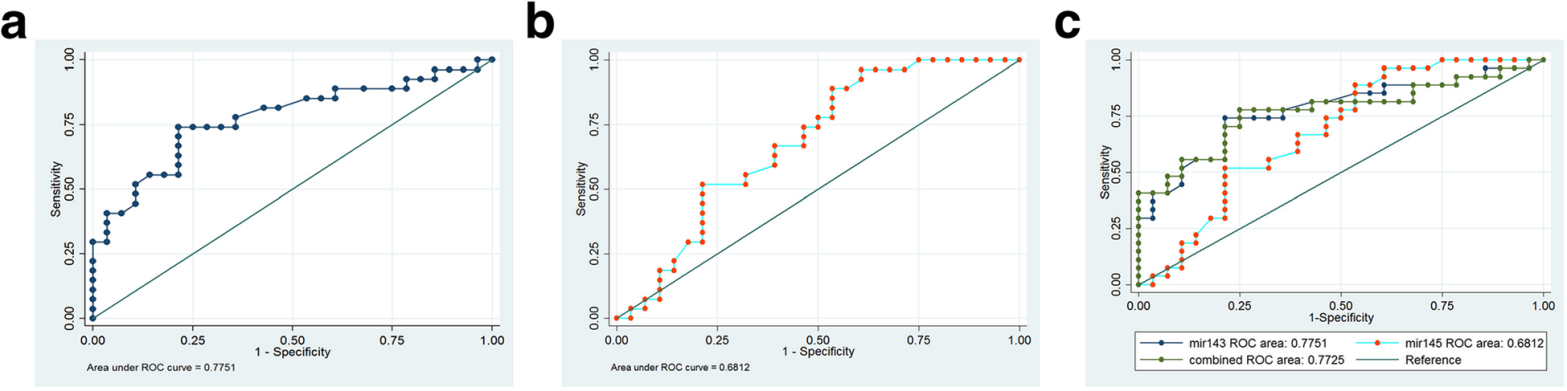
ROC curve for plasma (**a**) miR-143, (**b**) miR-145, and (**c**) the combination of the two miRNAs were able to distinguish all Hhcy patients (Hhcy + ATH and Hhcy) from the (NC and ATH) cases

**Fig. 5:**
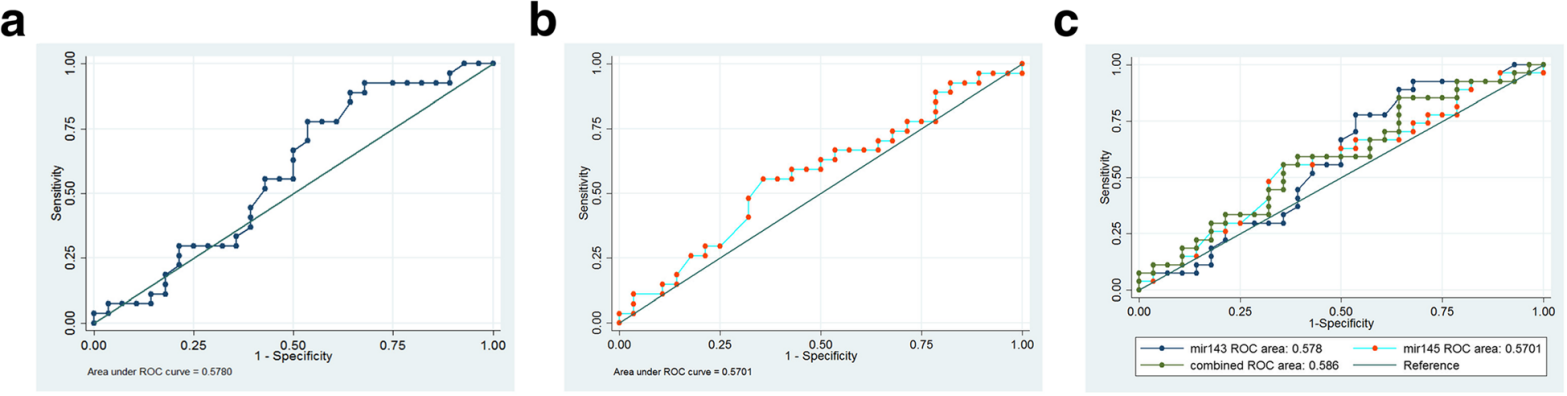
ROC curve for plasma (**a**) miR-143, (**b**) miR-145, and (**c**) the combination of the two miRNAs were able to distinguish all atherosclerosis patients (Hhcy + ATH and ATH) from the (NC and Hhcy) cases

To further evaluate the predictive power of atherosclerosis-associated miR-143 and miR-145 for Hhcy. All those subjects were divided into hyperhomocysteinaemia patient (34 men and 21 women; mean age 48.16 ± 5.51 years) and atherosclerosis patient (35 men and 20 women; mean age 48.15 ± 5.36 years) groups, and ROCcurves were constructed to estimate the sensitivities and specificities of miR-143/miR-145 levels for the detection of Hhcy and ATH patients. As shown in Fig. 4a and b, ROC curve analysis of miR-143 or miR-145 exhibited strong differentiation power between Hhcy patients and other groups (NC and ATH). The AUC of miR-143 or miR-145 in Hhcy patients was 0.775 (*P* < 0.001), 0.681 (*P* < 0.001). Interestingly, the combination of the two miRNAs resulted in a little lower AUC value of 0.773 (*P* < 0.001) than the AUC of miR-143 or miR-145 (Fig. 2a–c). These data suggested that the combination of circulating miR-143 and miR-145, which were equal to the sensitivity and specificity of miR-143 alone for diagnosing Hhcy. In contrast, the miR-143 and miR-145 levels did not significantly aid the detection of between ATH patients and other groups (NC and Hhcy) (Fig. 5a–c).

## Discussion

The present study is the only study to demonstrate the link between atherosclerosis-associated miR-143 and miR145 and hyperhomocysteinaemia in humans. In this study, all the subjects involved were freshly diagnosed with Hhcy and had never undergone any intervention whatsoever. Our study identified miR-143 and miR-145 as potential non-invasive biomarkers for Hhcy, which may be helpful in predicting the progress of atherosclerosis in Hhcy patients.

In this study, Hcy was positively correlated with TC and LDL-c, which indicates that elevated serum homocysteine might be closely related to dyslipidaemia and aggravate atherosclerosis. Meanwhile, qRT-PCR results showed that plasma miR-143 and miR-145 were visibly down-regulated in Hhcy + ATH patients. Pearson’s correlations revealed that the miR-143/−145 expression levels were negatively correlated with Hcy, TC, LDL-c or TG. The ROC analyses showed that the miR-143 might be suitable diagnostic markers for the detection of all Hhcy patients, including Hhcy and Hhcy + ATH patients.

Several studies have demonstrated that miR-143/−145 are molecular keys that determine VSMC phenotypic switching and are essential for VSMC differentiation [12], which is known to contribute to atherogenesis [31–33]. We found that that high level of LDL-c and Hcy are most important risk factor for atherosclerosis. And our data shows that miR-143/−145 expression level was negatively correlated with Hcy and LDL-c. Our data support the notion that Hcy was positively correlated with LDL-c. Several studies have demonstrated that elevated Hcy makes the correlation with LDL-c [34, 35]. This study demonstrated that elevated homocysteine levels may increase the risk of atherosclerosis.

The role of homocysteine in vascular plaque formation is multifactorial and includes smooth muscle proliferation, endothelial dysfunction and inflammation [36]. Previous studies have also confirmed the connection between Hhcy and dyslipidaemia, which is consistent with the results of present study [37]. The down-regulations of miR-145 and miR-143 in injured or atherosclerotic vessels are associated with proliferating, less differentiated smooth muscle cells [38]. Interestingly, miR-143/−145 are significantly down-regulated in clinical atherosclerotic plaque arteries compared with healthy arteries, which suggests that increasing miR-143 and miR-145 levels might contribute to the prevention of atherosclerotic plaque formation.

Because miR-143 and miR-145 are expressed in VSMCs, endothelial cells and inflammatory cells, it is not surprising that several animal and clinical studies have already demonstrated that miR-143 and miR-145 contribute to the pathogenesis of atherosclerosis [33, 39, 40]. MiR-145 has been found to be down-regulated in the proliferative VSMCs of atherosclerotic arteries in ApoE-knockout mice [41]. This finding implies that the down-regulation of miR-145 may contribute to atherogenesis. Hai Gao et al. [41] demonstrated that the plasma level of miR-145 is significantly lower in CAD patients compared to healthysubjects. This finding reveals that circulating miR-145 has been demonstrated to be regulated during coronary atherosclerosis. These studies indicate that miR-145 is involved in vascular injury and the migratory activity of VSMCs. In our study, the expressions of miR-143 and miR-145 were found to be down-regulated in individuals with carotid artery plaques. The athero-protective roles of miR-143 and miR-145 may be attributed to their abilities to promote the contractile VSMC phenotype and inhibit the synthetic VSMC phenotype, the latter of which is associated with atherosclerosis [6, 14].

MiRNA-based therapy has been regarded as a promising method for clinical applications in the treatment of cardiovascular diseases [42, 43]. Targeting miR-143/−145 may be a promising therapy for these cardiovascular diseases. However, to date, no miRNAbased therapy has been developed to treat cardiovascular diseases in human clinical trials. Several animal studies have demonstrated that targeting miR-143/−145 may be a promising therapy for vascular diseases. Our data just indicate that miR-143/−145 may be potential non-invasive markers of atherosclerosis in Hhcy patients, and our results may be impetus for these circulating miRNAs as prognostic biomarkers before long and possibly as therapeutic targets of atheroscleorsis in Hhcy patients.

Remarkably, the plasma microRNA levels were not affected by a wide range of clinical confounders, including age, sex, body mass index, kidney function, hepatic function and fasting blood glucose level.

The current study not only corroborated previous animal studies of the effect of hyperhomocysteinaemia on atherosclerosis but also linked atherosclerosis-associated microRNAs and hyperhomocysteinaemia in humans. This relationship provides novel insight into the pathophysiology of atherosclerosis. Ourstudy has some limitations. First, the mechanisms of miR-143/−145 in regulating atherosclerosis are still not entirely clear in Hhcy human and unexplored in this study, while the underlying mechanisms postulated were based on previous studies. Further experimental studies are needed to explore unknown functions of miR-143/−145 that was expressed in Hhcy patients. Carotid intima-media thickness (CIMT) could not be applied as an evaluation index for atherosclerosis due to a lack of equipment in the hospital. Another potential limitation of our study is the small number of patients available due to the low prevalence of hyperhomocysteinaemia.

## Conclusion

In conclusion, we demonstrated that atherosclerosis-related circulating miR-143/miR-145 have significantly variable expressions between NC, Hhcy, Hhcy + ATH and ATH individuals and between carotid plaque and no-plaque individuals. This study revealed that the miR-143/−145 expression levels were positively associated with Hcy and lipidaemia. Moreover, this study indicated that miR143may be regarded as a potential non-invasive biomarkers of atherosclerosis in patients with hyperhomocysteinaemia. However, prospective large-scale studies are required to determine the potential value of circulating miRNAs for the determination of atherosclerosis in patients with hyperhomocysteinaemia.

## Additional files

Additional file 1: Figure S1. Pearson’s correlation was used to explore the relationships between Hcy with TC and LDL. *P* < 0.05 or *P* < 0.001 was considered significant. (TIFF 57 kb)

Additional file 2: Figure S2. Pearson’s correlation was used to explore the relationships between miR-143 with Hcy, TC and LDL-c. *P* < 0.05 or *P* < 0.001 was considered significant. (TIFF 79 kb)

Additional file 3: Figure S3. Pearson’s correlation was used to explore the relationships between miR-145 with Hcy, TC, LDL and TG. *P* < 0.05 or *P* < 0.001 was considered significant. (TIFF 94 kb)

## Abbreviations

ALT: Alanine aminotransferase
ApoA: Apolipoprotein A; Apo A/B, apolipoprotein A/B
ApoB: Apolipoprotein B
AST: Aspartate aminotransferase
ATH: Carotid artery atherosclerosis alone subjects
BMI: Body mass index
Cre: Serum creatinine
FBG: Fasting blood glucose
Hcy: Homocysteine
HDL-c: High-density lipoprotein cholesterol
Hhcy: Hyperhomocysteinaemia alone subjects
Hhcy + ATH: Hyperhomocysteinaemia and carotid artery atherosclerosis combined subjects
LDL-c: Low-density lipoprotein cholesterol
NC: Normal control subjects
TC: Total cholesterol
TG: Triglyceride
UA: Uric acid
