# Supplementary figures and images for: Expression levels of atherosclerosis-associated miR-143 and miR-145 in the plasma of patients with hyperhomocysteinaemia
Source: BMC Cardiovasc Disord. 2017 Jun 20;17:163. doi: 10.1186/s12872-017-0596-0 (PMC5477732; doi:10.1186/s12872-017-0596-0)

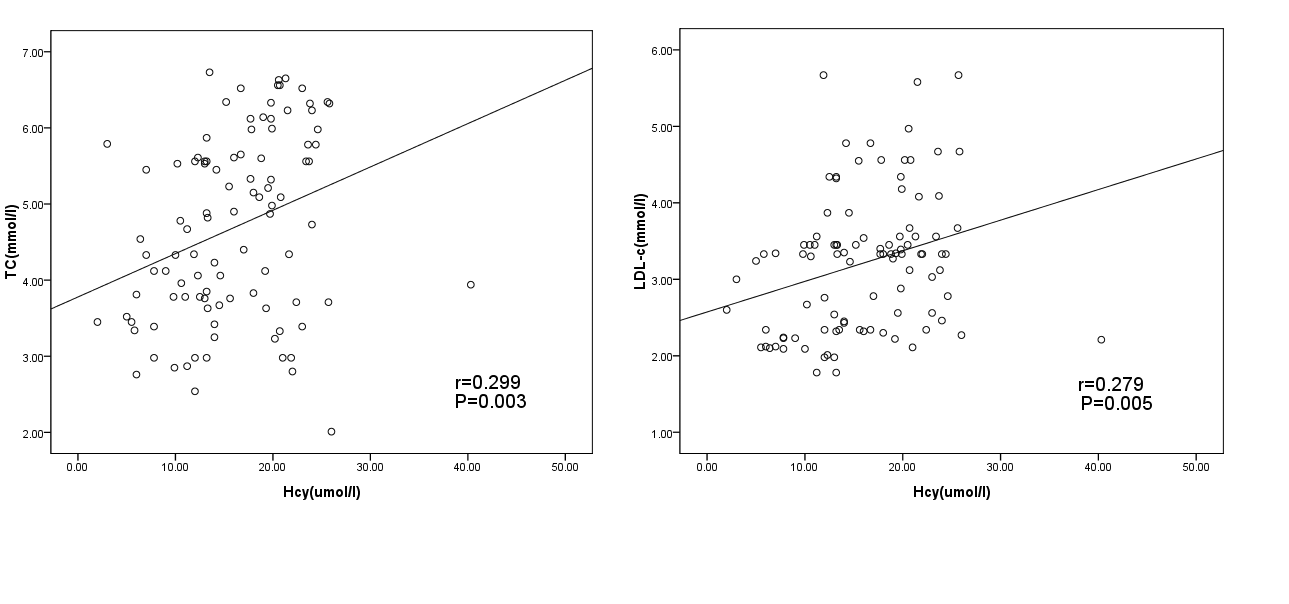

Supplement: Supplementary file 1 — Pearson’s correlation was used to explore the relationships between Hcy with TC and LDL. P < 0.05 or P < 0.001 was considered significant. (TIFF 57 kb) [file 12872_2017_596_MOESM1_ESM.tif]

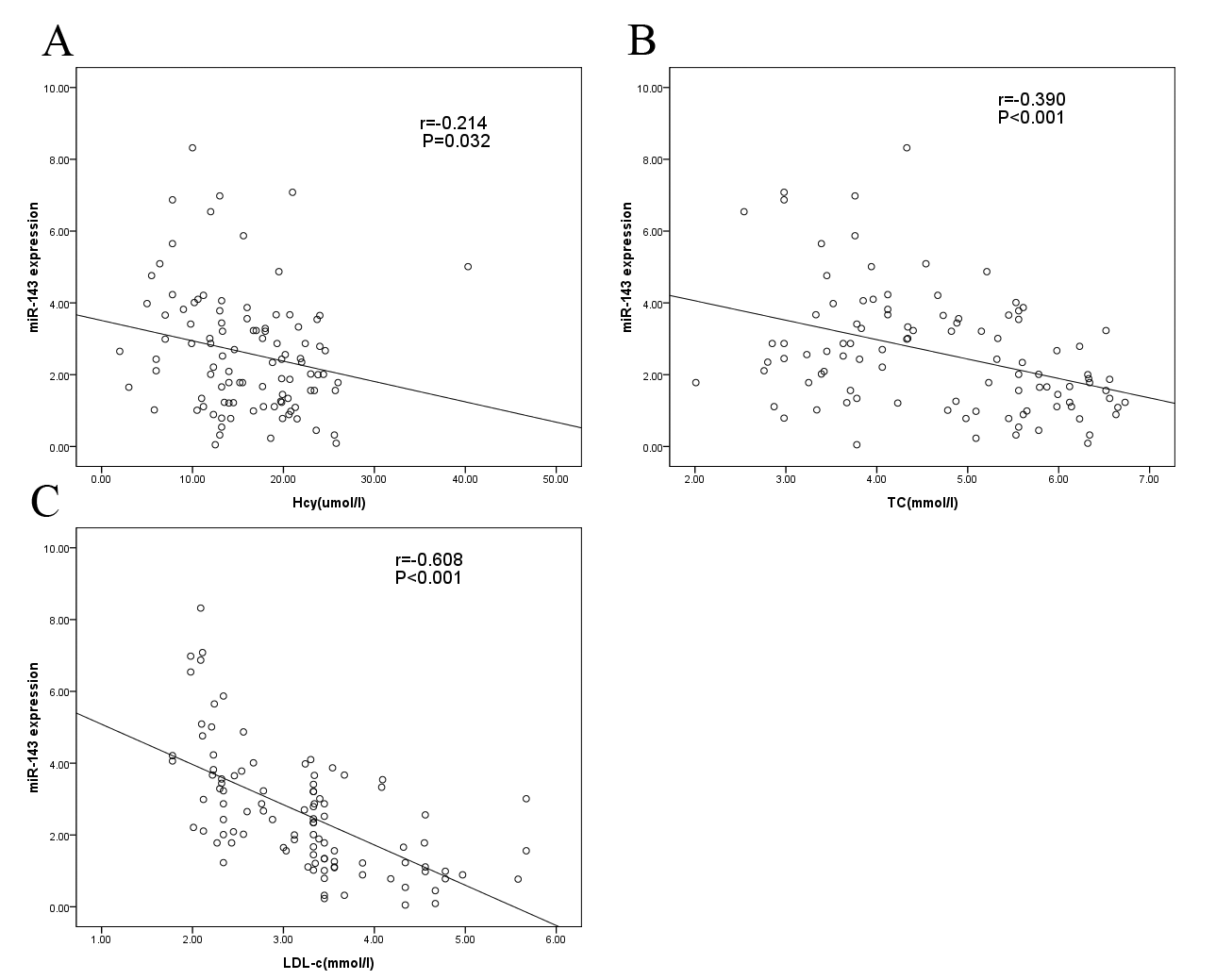

Supplement: Supplementary file 2 — Pearson’s correlation was used to explore the relationships between miR-143 with Hcy, TC and LDL-c. P < 0.05 or P < 0.001 was considered significant. (TIFF 79 kb) [file 12872_2017_596_MOESM2_ESM.tif]

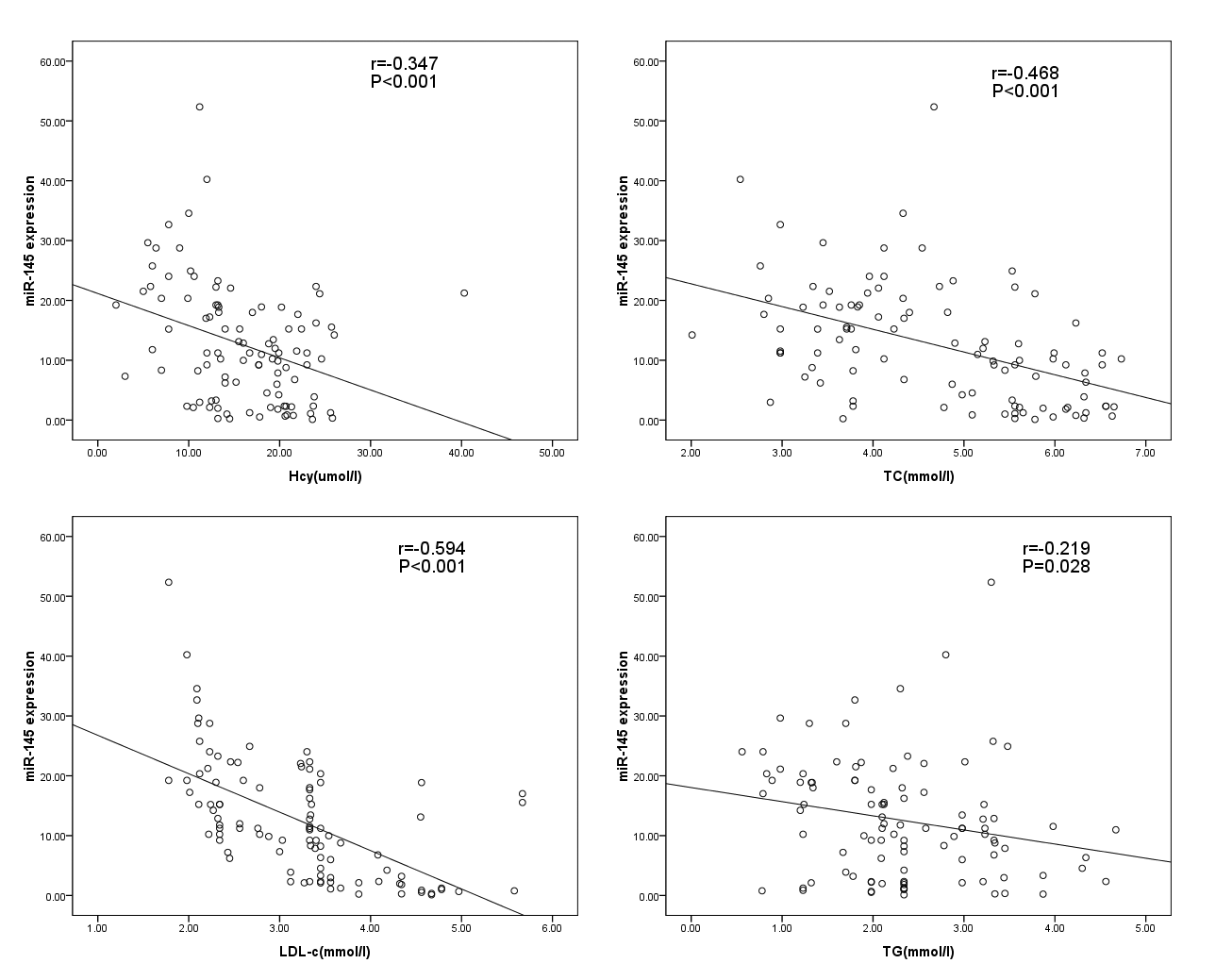

Supplement: Supplementary file 3 — Pearson’s correlation was used to explore the relationships between miR-145 with Hcy, TC, LDL and TG. P < 0.05 or P < 0.001 was considered significant. (TIFF 94 kb) [file 12872_2017_596_MOESM3_ESM.tif]
